# Rolling Bearing Performance Degradation Assessment with Adaptive Sensitive Feature Selection and Multi-Strategy Optimized SVDD

**DOI:** 10.3390/s23031110

**Published:** 2023-01-18

**Authors:** Zhengjiang Feng, Zhihai Wang, Xiaoqin Liu, Jiahui Li

**Affiliations:** 1 Key Laboratory of Advanced Equipment Intelligent Manufacturing Technology of Yunnan Province, Kunming University of Science & Technology, Kunming 650500, China; 2 Faculty of Mechanical & Electrical Engineering, Kunming University of Science & Technology, Kunming 650500, China

**Keywords:** performance degradation assessment, rolling bearing, SVDD, feature selection, multi-strategy optimization

## Abstract

In light of the problems of a single vibration feature containing limited information on the degradation of rolling bearings, the redundant information in high-dimensional feature sets inaccurately reflecting the reliability of rolling bearings in service, and assessments of the degradation performance being disturbed by outliers and false fluctuations in the signal, this study proposes a method of assessing rolling bearings’ performance in terms of degradation using adaptive sensitive feature selection and multi-strategy optimized support vector data description (SVDD). First, a high-dimensional feature set of vibration signals from rolling bearings was extracted. Second, a method combining the Technique for Order Preference by Similarity to an Ideal Solution (TOPSIS) and K-medoids was used to comprehensively evaluate the features with multiple evaluation indicators and to adaptively select better degradation features to construct the sensitive feature set. Next, multi-strategy optimization of the SVDD model was carried out by introducing the autocorrelation kernel regression (AAKR) and a multi-kernel function to improve the ability of the evaluation model to overcome outliers and false fluctuations. Through validation, it could be seen that the method in this study uses samples of rolling bearings in the healthy early stage to establish the evaluation model, which can adaptively determine the starting point of the bearing’s degradation. The stability and accuracy of the model were effectively improved.

## 1. Introduction

The failure of rolling bearings, as one of the key components of rotating machinery, leads to the breakdown of the whole mechanical system [1,2]. During the in-service period, the performance of rolling bearings degrades irreversibly due to fatigue, wear, and other reasons. Effective assessment in the performance degradation assessment (PDA) of rolling bearings in the service phase can help organize maintenance in a targeted manner to prevent failure from occurring and improve the operational reliability of the whole machine.

Assessment of the degradation in the performance of rolling bearings mainly includes three steps: acquisition of the rolling bearings’ monitoring data, feature extraction, and establishment of the model for assessing the degradation. The degradation mechanism of rolling bearings is complex, and the vibration signals of rolling bearings are nonlinear and nonstationary. A single feature contains less information about bearings degradation and has poor anti-interference ability, so it cannot accurately characterize the whole process of degradation during performance. Constructing a high-dimensional feature set can comprehensively reflect the information on the bearings’ degradation and benefit from the complementarity of the differences among features, but some features are unrelated to degradation. Irrelevant features can be eliminated using feature selection methods, which can be classified into two categories based on whether the methods are independent of subsequent learning algorithms, namely filters and wrappers [3]. The wrapper method is computationally complex and less versatile because it requires several iterations in combination with subsequent learning algorithms to find the best combination of features. Feature evaluation [4,5] is one of the commonly used filtering methods and is independent of the subsequent learning algorithm. It can quickly remove irrelevant features with high generality and interpretability, so it is often used in engineering applications [6]. Although feature ranking can be achieved using feature evaluation, the selection of the feature set relies heavily on experts’ prior knowledge, which reduces the efficiency of the algorithm and may introduce subjective errors.

In general, data-driven fault diagnosis techniques use machine learning algorithms to identify fault status to train predictive models based on the condition data collected under normal and different faulty states [7]. The training of the model is based on the condition data collected in the health stage and the degradation stage. Most of these data-driven approaches rely entirely on data at different stages [8,9]. For assessing the degradation in the performance of rolling bearings, the process of bearing degradation usually consists of the healthy phase and the degradation phase. In actual production, fewer data are available on the degradation stage, and sometimes, only data from the healthy state are available. Knowledge-based methods often need to capture a large number of samples in advance to identify faults [10]. Rai et al. [11] performed K-medoids clustering to train a model using the full-life feature set of bearings obtained with empirical modal decomposition (EMD) and calculated the dissimilarity between the bearing samples to be tested and the clustering centers used as health indicators. Pan et al. [12] used lifting wavelet packet decomposition and fuzzy c-means combined with the affiliation function to characterize the severity of bearings failure. Adaptive determination of the start time of the degradation phase (first predicting time, FPT) can effectively trigger an early warning to carry out condition-based maintenance. Heng et al. [13] used principal component analysis (PCA) to fuse the time domain features to extract the life cycle health index of rolling bearings and then divided the performance stages according to the amplitude of the change trends of vibration signals. These methods are suitable for obtaining health indicators but ignore the difficulties of obtaining data from the degradation stage and carrying out secondary determination of FPT. Finally, the data monitoring process inevitably suffers from the interference of noise and environmental changes, which lead to outliers and false fluctuations in the data. Liu et al. [14] used the features extracted from the time domain combined with the SVDD model to monitor the faults in rolling bearings and to overcome the interference of random fluctuations by using a decision strategy. The authors of [15] used the method of repairing the evaluation results, which created problems such as subjectivity and reduced interpretability. The recognition ability and robustness of models in mechanical learning are always required [16,17]. Therefore, there are still shortcomings in using sensitive feature sets for a PDA of bearings, such as the models’ reliance on data for the full life cycle of the bearings, FPT needing to be determined twice, and the evaluation model being easily affected by outliers and false fluctuations. How to achieve efficient fault diagnosis using only health data has attracted our attention.

In summary, extracting effective feature sets is a prerequisite for accurately assessing the performance of bearings, and improving the ability of the model to overcome outliers and false fluctuations is one of the critical tasks in assessing degradation. Accordingly, a rolling bearing performance degradation assessment method with the combination of adaptive sensitive feature selection and multi-strategy optimized SVDD was proposed in this paper. The specific contributions of this study are described below. TOPSIS-Kmedoids, an adaptive sensitive feature selection method, was proposed, which could determine the adaptive sensitive feature set without prior knowledge. In addition, SVDD was optimized using a multi-strategy, in which AAKR was introduced to correct the errors in monitoring data, and a multi-kernel function was constructed to improve the learning ability and generalization ability of the model. Lastly, the effectiveness of the proposed method was verified using the XJTU-SY dataset for the full life cycle of rolling bearings from Xi’an Jiaotong University, the PHM2012 Data Challenge dataset for the full life cycle of rolling bearings, and a set of data from a self-made bench test of accelerated fatigue in rolling bearing.

## 2. Determination of the Adaptive Sensitive Feature Set

### 2.1. Feature Extraction

In the field of prognostics and health management (PHM) of rolling bearings, the vibration signal is one of the most commonly used means because it contains much information on degradation. Feature extraction can reveal information on the performance of sensor data. Twenty-four commonly used statistical features of vibration were extracted from the time domain and the frequency domain of vibration signals, as shown in Table 1, where *F*_1_–*F*_7_ are the frequency domain features, $s_{i}$ is the amplitude of the vibration data, $sk_{i}$ is the spectral amplitude of the vibration data, and $f_{i}$ is the frequency of the vibration data. For the data from two accelerometers, the features listed in Table 1 were extracted separately to form the high-dimensional set, where *n* is the feature length and *m* is the number of features.

### 2.2. Feature Evaluation with Multiple Criteria

The quality of features significantly affects the results when assessing the degradation. Good features should correlate strongly with the bearings’ degradation process with monotonic increasing or decreasing characteristics and robustness to outliers [3]. Most of the existing methods for evaluating the quality of features use a single-indicator evaluation scheme. To select excellent features, this study simultaneously considered the three indicators of monotonicity, correlation, and robustness [18], which are described as follows:

(1) Monotonicity: The degradation of rolling bearings’ performance is an irreversible process, so the features should be able to monotonically characterize the process of rolling bearings from operation to failure. Because the time vector $t_{i}$ is strictly monotonic, the correlation coefficient $Mon(A_{i})$ between the feature vector $A_{i}$ and the time vector $t_{i}$ is used to measure the monotonicity of the feature [19]. In practice, rolling bearings often show a nonlinear degradation trend. The Spearman’s rank correlation coefficient is widely applicable and is more sensitive to nonlinear correlation [1], so this study calculated the Spearman’s rank correlation coefficient as the monotonicity index of the feature. The monotonicity score’s equation is shown in Equation (1) [3] as:

(1) $$Mon(A_{i})=1-\frac{6\times \sum_{i=1}^{n}{\left[rank(A_{i})-rank(t_{i})\right]}^{2}}{n(n^{2}-1)}$$

where $rank(A_{i})$ and $rank(t_{i})$ indicate $A_{i}$ and $t_{i}$ in ascending order, respectively, and *n* is the feature length.

(2) Robustness: During the use of rolling bearings, the signal acquisition process is inevitably disturbed by the environment, changes in the working condition, and noise. The robustness index $Rob(A_{i})$ is used to measure the tolerance of the features to random noise and abnormal values [19]. Equation (2) is used for calculating the robustness score of features, which is a widely used and interpretable equation for calculating the robustness index of features [1], as follows:

(2) $$Rob(A_{i})=\frac{\sum_{i=1}^{n}\exp(-|f_{i}^{r}/A_{i}|)}{n}$$

where $f_{i}^{t}$ and $f_{i}^{r}$ are the trend and residual values of the *i*th feature $A_{i}$, respectively. These two items can be obtained using smoothing methods and satisfying the equation $A_{i}=f_{i}^{t}+f_{i}^{r}$ [3].

(3) Correlation: The correlation index $Cor(A_{i})$ is used to measure whether the feature can capture the trend of the degradation in performance across the life cycle of rolling bearings [20]. The equation for calculating the correlation score is shown in Equation (3), which can measure the change trend of the features across the whole life cycle [3], as:

(3) $$Cor(A_{i})=\frac{\left|n\sum_{i=1}^{n}\left(if_{i}^{t}\right)-\sum_{i=1}^{n}f_{i}^{t}\sum_{i=1}^{n}i\right|}{\sqrt{\left[n\sum_{i=1}^{n}{\left(f_{i}^{t}\right)}^{2}-(\sum_{i=1}^{n}f_{i}^{t}{)}^{2}]-[n\sum_{i=1}^{n}i^{2}-{\left(\sum_{i=1}^{n}i\right)}^{2}\right]}}$$

where $f_{i}^{t}$ is the trend values of the *i*th feature $A_{i}$.

All the indexes above are positive indicators; that is, the higher the evaluation score, the better the feature’s quality. 

### 2.3. Adaptive Sensitive Feature Selection

**Figure 1:**
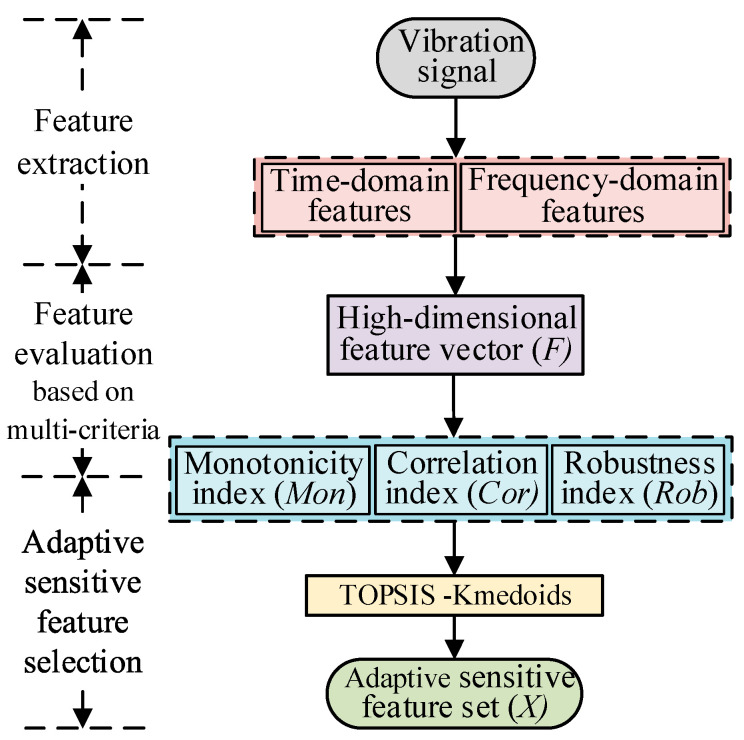
Process for selecting the adaptive sensitive features.

A single metric can barely make a comprehensive and accurate evaluation of the degradation features. Sensitive features should be selected using integration of the evaluation indicators mentioned in Section 2.2. Linear weighting is commonly used to construct the comprehensive metrics when relying on multiple metrics to evaluate features, and the allocation of weights will directly impact the results of evaluation. The Technique for Order Preference by Similarity to Ideal Solution (TOPSIS) method is a commonly used comprehensive evaluation method that constructs comprehensive metrics without relying on prior knowledge to subjectively determine weights [3]. Meanwhile, the selection of sensitive feature sets has the disadvantage of relying on the prior knowledge of experts. The K-medoids algorithm is a robust clustering algorithm, which can divide features into clusters according to rules to realize adaptive classification of the features. Therefore, the TOPSIS–K-medoids method was applied for a comprehensive evaluation of the features with the evaluation indexes constructed using Equations (1)–(3) and for constructing the adaptive sensitive feature set. The key steps are shown in Figure 1 and described below. 

Step 1: Construction and normalization of the evaluation matrix. The feature evaluation matrix $Q=[Mon(A_{i});Cor(A_{i});Rob(A_{i})]$ is constructed using Equations (1)–(3), where M×N is a matrix of Q dimensions, M is the number of features (*M* = 48 in this study), and N is the number of evaluation metrics (*N* = 3 in this study). The evaluation matrix is normalized using Equation (4):

(4) $$y_{ij}=q_{ij}/\sqrt{\sum_{i=1}^{M}q_{ij}{}^{2}}$$

where $q_{ij}$(1 ≤ i ≤ *M*, 1 ≤ j ≤ *N*) represents the elements in the evaluation matrix Q and  denotes the standardized value of the *j*th evaluation metric for the *i*th feature.

Step 2: Calculation of the TOPSIS score. The maximum and minimum values of each evaluation index are obtained and defined as the superior solutions $Y_{j}^{+}$ and inferior solutions $Y_{j}^{-}$, then the TOPSIS scores $S_{i}$ of the features are calculated according to Equation (5) [4]:

(5) $$S_{i}=\sqrt{\sum_{i=1}^{M}{\left(Y_{j}^{-}-y_{ij}\right)}^{2}}/[\sqrt{\sum_{i=1}^{M}{\left(Y_{j}^{+}-y_{ij}\right)}^{2}}+\sqrt{\sum_{i=1}^{M}{\left(Y_{j}^{-}-y_{ij}\right)}^{2}}]$$

where the TOPSIS score is positively correlated with the signal, such that the higher the score $S_{i}$, the richer the degradation information contained in the feature.

Step 3: Feature clustering. The K-medoids algorithm was improved from the K-means method. K-medoids is a clustering algorithm with good robustness. To achieve adaptive classification of the features, K-medoids is used to divide the data into class clusters according to certain rules, so that samples of the class cluster are similar. In order to adaptively determine the sensitive features set, the feature selection process is transformed into the K-medoids clustering problem with the TOPSIS score. The core idea of the K-medoids algorithm is to divide the feature scores, as obtained in Step 2, into clusters under the condition that the sum of dissimilarities between the cluster’s elements and the cluster’s center is minimized [6]. The highest cluster is then extracted and determined to be the sensitive feature set. The sum of algorithmic dissimilarities *J* is calculated as follows:

(6) $$\min J=\sum_{j=1}^{k}\sum_{x_{i}=c_{j}}^{}D(S_{i},o_{j})$$

where $c_{j}$ is the *j*th cluster, $o_{j}$ is the *j*th medoid, and $D(S_{i},o_{j})$ is the distance between $S_{i}$ and $o_{j}$. To overcome the problem that the K-medoids clustering algorithm can easily fall into the local optimal state because of improper initial point selection, selecting samples with the relative distance as the initial clustering center can effectively improve this situation. A trade-off between similarity and the weighted Euclidean distance can improve the classification’s accuracy [20]. Therefore, the improved K-medoids clustering algorithm selects the initial clustering center with a more considerable distance and divides the features into clusters according to the similarity of the weighted Euclidean distances. The weight is the proportion of the feature’s score to the sum of all features’ scores, and the weighted distance calculation equation is:

(7) $$D(S_{i},o_{j})=∥S_{i}-o_{j}{∥}^{2}(S_{i}/\sum_{i=1}^{n}S_{i})$$

Step 4: Adaptive sensitive feature set. The medoid is adjusted using iteration according to Equation (6) until the center point no longer changes. The cluster with a large TOPSIS value of M in the center of the cluster is determined as the adaptive sensitive feature set $X^{L\times m}$, where *L* is the length of the sensitive features, m is the number of selected features, and $x_{i}$ is the *i*th sensitive feature.

## 3. Multi-Strategy Optimized Support Vector Data Description

### 3.1. Support Vector Data Description

**Figure 2:**
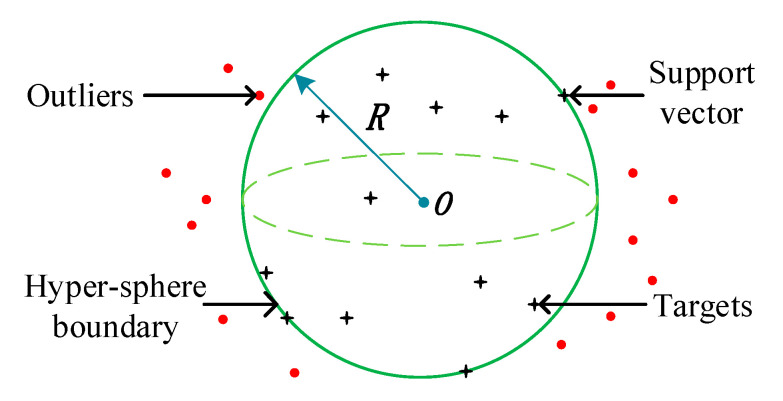
Schematic diagram of SVDD.

SVDD is an effective one-class classification algorithm proposed by Tax et al. [21] in 1999. The core idea of the SVDD algorithm is as follows: The target samples are first mapped to the high-dimensional feature space using nonlinear transformation, then a minimum hypersphere containing most, if not all, the training samples is established in the feature space. In contrast, the nontarget values are distributed outside the hypersphere as much as possible [13]. SVDD uses the hypersphere as its decision surface, and a schematic diagram of this is shown in Figure 2. The center O and the radius R of the sphere are the decision variables of the hypersphere, the samples on the boundary of the hypersphere are the support vectors, and the samples outside the hypersphere are outliers. 

SVDD is a semi-supervised model in which only one type of target sample is required for the model’s training, that is, the model can be trained with samples from normal stages. The labeled samples are the training set of SVDD, which needs to construct the minimum radius hypersphere. The objective function can be described using Equation (8):

(8) $$F(R,O)=\min R^{2}+C\sum_{i=1}^{n}\xi_{i}$$

The objective function must also satisfy the constraints $∥x_{i}-o∥\leq R^{2}+\xi_{i}$, $\xi_{i}\geq 0$, where $x_{i}$ represents the labeled samples; $\xi_{i}$ is the relaxation variable, which allows a small number of labeled samples to be distributed outside the hypersphere to reduce the effect of outliers on the radius of the hypersphere; and C is the penalty coefficient, which acts to maintain the balance between the size of the radius *R* and the number of samples falling outside the sphere.

Equation (8) is a quadratic convex optimization problem with univariate variables. By introducing Lagrangian multipliers, constraints are fused into the objective functions to form dual forms, and the following results can be obtained:

(9) $$\max L=\sum_{i=1}^{n}\alpha_{i}\left(x_{i}\cdot x_{i}\right)-\sum_{i,j=1}^{n}\alpha_{i}\alpha_{j}\left(x_{i}\cdot x_{j}\right)$$

where $x_{j}$ represents the labeled samples, and $\left(x_{i}\cdot x_{j}\right)$ is the inner product of $x_{i}$ and $x_{j}$.

To cope with the nonlinearity problem, the kernel function is introduced to replace the inner product. The kernel function maps the data to the high-dimensional feature space, which makes the nonlinear data easier to linearly separate in the high-dimensional feature space. According to the Karush–Kuhn–Tucher condition [22], it is known that the training samples satisfying $\alpha_{i}=0$ will be wrapped inside the hypersphere; those satisfying $0<\alpha_{i}<C$ will be the support vector; and those with $\alpha_{i}=C$ are judged to be outliers. The radius of hypersphere is obtained as follows:

(10) $$R={\left[K\left(x_{i}\cdot x_{i}\right)-2\sum_{i=1}^{n}\alpha_{i}K\left(x_{i}\cdot x_{j}\right)+\sum_{i,j=1}^{n}\alpha_{i}\alpha_{j}K\left(x_{i}\cdot x_{j}\right)\right]}^{1/2}$$

where $K\left(x_{i}\cdot x_{i}\right)$ is the kernel function. 

The equation for calculating the distance between the new sample $x_{n}$ and the center R of the hypersphere is:

(11) D=[K(xn⋅xn)−2∑i=1nαiK(xi⋅xn)+∑i,j=1nαiαjK(xi⋅xj)]12

### 3.2. Construction of the Multi-Kernel Function

Single-kernel functions have limitations in dealing with outliers and false fluctuations. Different kernel functions have different levels of efficacy, and multiple kernel functions combine different types of kernel functions, which can combine good learning ability and generalization. Using multi-kernel functions can make the results of SVDD more robust [23,24]. The methods of constructing multi-kernel functions include multi-scale kernels and synthetic kernel functions. The synthetic kernel approach has high learning ability and generalization ability and low operational complexity. Therefore, this method was used to construct multi-kernel functions as follows:

(12) $$K_{m}(x_{i},x_{j})=\omega K_{1}(x_{i},x_{j})+(1-\omega )K_{2}(x_{i},x_{j})$$

where $K_{m}(x_{i},x_{j})$ represents the multi-kernel functions; ω represents the weights, 0<ω<1; and $K_{1}(x_{i},x_{j})$ and $K_{2}(x_{i},x_{j})$ are single-kernel functions.

The single-kernel functions include Gaussian, Sigmoid, and Laplace kernel functions, and the kernel functions are calculated using:

(13) $$\left\{ \begin{array}{l} k_{Gauss}(x_{i},x_{j})=\exp(-∥x_{i}-x_{j}{∥}^{2}/\sigma_{1}^{2})\\ k_{\mathrm{Tanh}}(x_{i},x_{j})=\tanh(\sigma_{2}x_{i}{}^{T}x_{j}+\sigma_{3})\\ k_{Lapl}(x_{i},x_{j})=\exp(-∥x_{i}-x_{j}{∥}^{2}/\sigma_{4}) \end{array}\right.$$

where $\sigma_{1}$, $\sigma_{2}$, $\sigma_{3}$, and $\sigma_{4}$ are the kernel parameters.

### 3.3. Auto-Associative Kernel Regression

The monitoring data of rolling bearings cannot avoid outliers and false fluctuations due to the interference of noise, working conditions, and environmental changes. These may greatly impact the performance of the model [25]. Using auto-associative kernel regression to reconstruct the signals can reduce the influence of outliers, so as to improve the robustness and recognition accuracy of the model [26,27]. AAKR was computationally efficient without relying on expert experience to adjust the parameters [3]. Therefore, AAKR was introduced to correct errors in the monitoring data by reconstructing the current feature matrix using the values of health history features to improve the evaluation ability of the models of degradation.

The core idea of AAKR is to map the characteristic matrix at the current moment to estimate the characteristic matrix of rolling bearings in the healthy state. AAKR maps the characteristic matrix at the current moment $X_{t}$ in the source space of degradation conditions to the data of the expected state ${\overset{\wedge }{X}}_{t}$ in the target space of normal conditions using:

(14) $${\overset{\wedge }{X}}_{t}=\sum_{i=1}^{m}\left(\omega_{i}\cdot X^{o}\right)/\sum_{i=1}^{m}\omega_{i}$$

where m is the number of optimal degradation features selected, $\omega_{i}$ represents the weights, and $\omega_{i}$ is determined by the similarity between $X^{o}$ and $X_{t}$. AAKR uses a Gaussian radial basis function as the kernel for mapping, and the values of $\omega_{i}$ are calculated as follows:

(15) $$\omega_{i}=e^{-di^{2}/2h^{2}}/\sqrt{2\pi h^{2}}$$

where h is the kernel’s bandwidth and $di^{2}$ is the distance between $X^{o}$ and $X_{t}$. Both distance similarity and spatial similarity were considered, and the equation is as follows:

(16) $$di^{2}=1+{\left(X_{t}-X^{o}\right)}^{T}S^{-1}(X_{t}-X^{o})-(X_{t}\cdot X^{o})/(|X_{t}|\cdot |X^{o}|)$$

where S is the diagonal matrix. The value of the diagonal line is the variance of the historical observation matrix.

### 3.4. Parameters of SVDD and Indicators of Degradation 

The parameters C, σ, and ω of SVDD were determined using particle swarm optimization. The fitness function of the minimum number of support vectors was used for SVDD parameter optimization [14,28], and the calculation equation Fit of fitness function is as follows:

(17) $$Fit=N_{SV}/N_{a}$$

where $N_{SV}$ is the number of support vectors; and $N_{a}$ is the total number of training samples. In order to ensure the robustness of SVDD, the minimum number of support vectors is set to 5% of the total number of training samples, and the optimization range of C is set to [1/$N_{a}$, 1/0.05$N_{a}$]. The optimization range of another parameter σ is [0.01, 10] [28]. The optimization range of another parameter ω is [0.01, 1].

To carry out a PDA of rolling bearings, the radius of the hypersphere was determined by carrying out multi-strategy optimized SVDD training with some of the previous normal samples. In the testing stage, the distance *D* between the test samples and the center of the hypersphere was calculated as the health indicator (HI) according to Equation (11). When D≤R, this indicated that the bearing was in the healthy stage; when D>R, this indicated that the bearing had degraded, and a larger value indicated that the degradation of the bearing was more serious. Moreover, when the HI exceeded the threshold value five times in a row, the point where the threshold value was exceeded for the first time was determined as the FPT.

## 4. Steps of the Algorithm for Assessing Degradation

**Figure 3:**
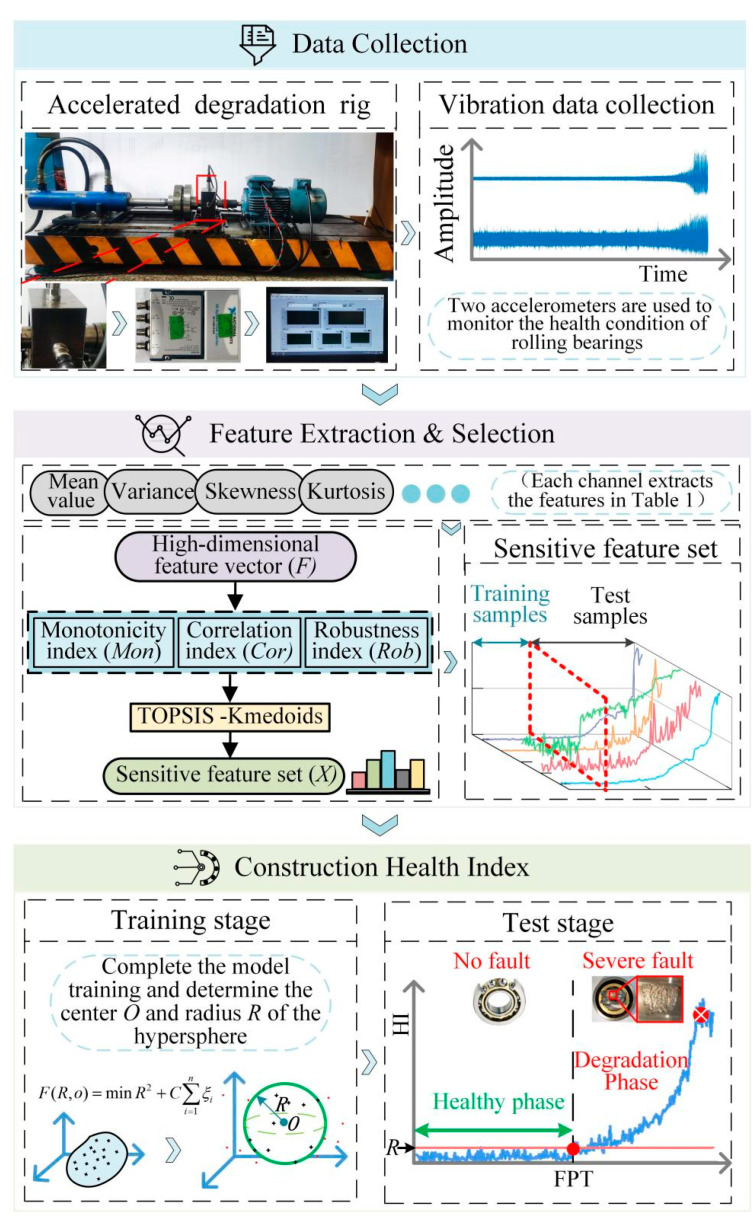
The technical process of the proposed method.

The process used for a PDA of rolling bearings with an adaptive sensitive feature set and multi-strategy optimized SVDD is shown in Figure 3. 

The main steps are as follows:

Step 1: Data acquisition. Obtain vibration signals of the rolling bearing degradation test platform. 

Step 2: Construction of the adaptive sensitive feature set. The multi-domain high-dimensional feature set A is constructed according to Table 1, and the adaptive sensitive feature set **X** is determined with the TOPSIS–K-medoids method described in Section 2.3.

Step 3: Optimize SVDD with multiple strategies. AAKR is introduced to correct the errors in the monitoring data, and a multi-kernel function is constructed to improve the learning ability and generalization ability of the model.

Step 4: Complete the training of the model. The samples in the early normal state of the sensitive feature set are taken as the training data and are determined to be the historical observation matrix. Training of the SVDD hypersphere using multi-strategy optimization is completed to obtain the hypersphere’s radius *R* and center *O.*

Step 5: Assessment of the degradation in performance. The test samples are inputted into the completed model, and the performance is evaluated according to the distance value *D* outputted by the model. Meanwhile, *R* is set as the adaptive alarm threshold. When D>R occurs several times in a row, the first occurrence point is determined as the FPT, and an early warning is given regarding the degraded state of the rolling bearings.

## 5. Experimental Verification

### 5.1. Case 1: XJTU-SY Bearing Datasets

#### 5.1.1. Experimental Description of Case 1

**Figure 4:**
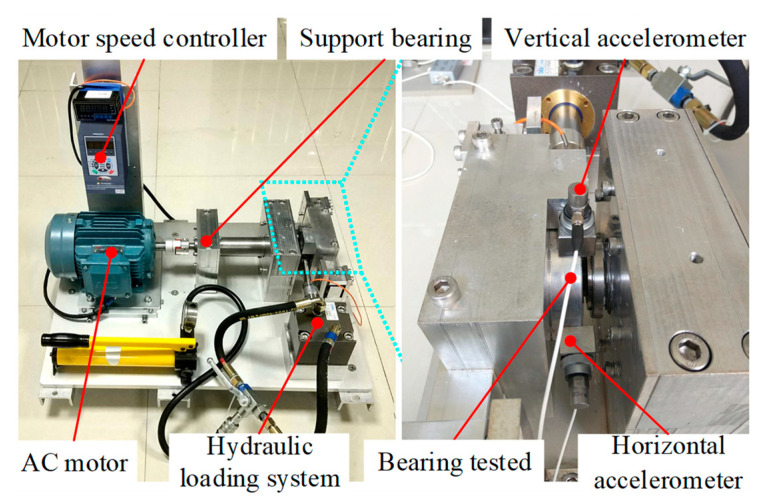
Platform used for the accelerated degradation test of the XJTU-SY bearing datasets.

**Figure 5:**
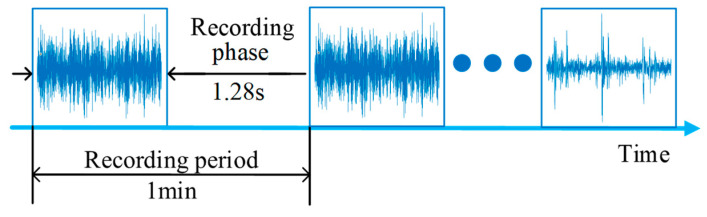
Illustration of the sampling parameters for the vibration signals.

The XJTU-SY rolling bearing dataset was used for verification. Figure 4 shows the platform used for the accelerated bearing degradation test in the experiment, which consisted of the test bearings, an AC motor, a controller of the motor’s speed, a hydraulic loading system, and other components [29]. Two accelerometers (model PCB352C33) were used to collect the horizontal and vertical vibration signals of the bearing across the entire life cycle, with a sampling frequency of 25.6 kHz. The signals were sampled for 1.28 s every 1 min during the experiment. The Illustration of sampling parameters is shown in Figure 5.

**Figure 6:**
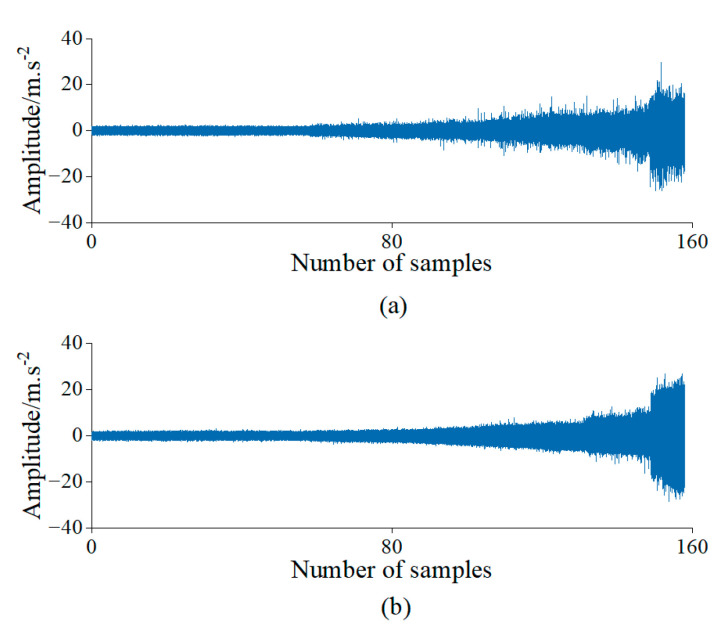
Bidirectional acceleration waveforms of rolling bearings in the time domain of Case 1. (**a**) Horizontal vibration signals; (**b**) Vertical vibration signals.

The subset Bearing 1–3 was used for the experiment and analysis, which collected 158 samples. Figure 6 shows the time domain waveform of the data for the bearings’ complete life cycle.

**Figure 7:**
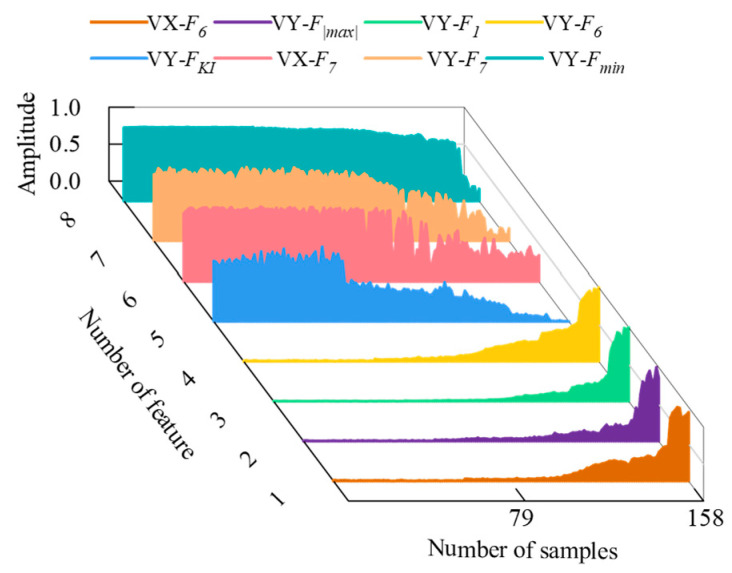
Sensitive features.

The PDA of rolling bearings was carried out according to the technical scheme in Figure 3. First, the time domain and frequency domain features of the vibration signal were extracted to construct a high-dimensional feature matrix and normalize it. Next, in order to remove the invalid features, the evaluation index of each feature was calculated, and the adaptive sensitive feature set was constructed according to Equations (4)–(7). The adaptive sensitive features thus determined are shown in Figure 7 (the notation VX-FX represents the corresponding features in Table 1 extracted from the vibration signal in the horizontal direction, and VY-FX represents the corresponding features from the vibration signal in the vertical direction). As the performance of the rolling bearing deteriorates, the sensitive features change according to different patterns. Each characteristic contains different information about the degradation of the rolling bearing. Finally, the samples from the early part of the healthy stage (i.e., the first 25% of all the samples) were selected as the training samples to complete the training of the SVDD model after multi-strategy optimization. Next, the test samples were fed into the model obtained using training, and the variation trend of the distance *D* from each sample to the center of the sphere was recorded. 

#### 5.1.2. Experimental Result of Case 1

To verify the effectiveness of the proposed method, commonly used degradation assessment methods were selected for comparison, including (a) constructing performance metrics by fusing the sensitive features with a PCA, (b) using root mean square (RMS) feature metrics, (c) combining a continuous hidden Markov model (CHMM) with the sensitivity to build assessment metrics, and (d) using the original SVDD combined with sensitive features to build assessment metrics. Method (d) and the proposed method could determine the warning threshold of degradation adaptively, and the rest of the methods determined the threshold using the three principles of the international engineering standard ISO-10816 [30]. When the degradation index exceeded the threshold five consecutive times, the first point where the threshold was exceeded was determined to be the FPT [31].

**Figure 8:**
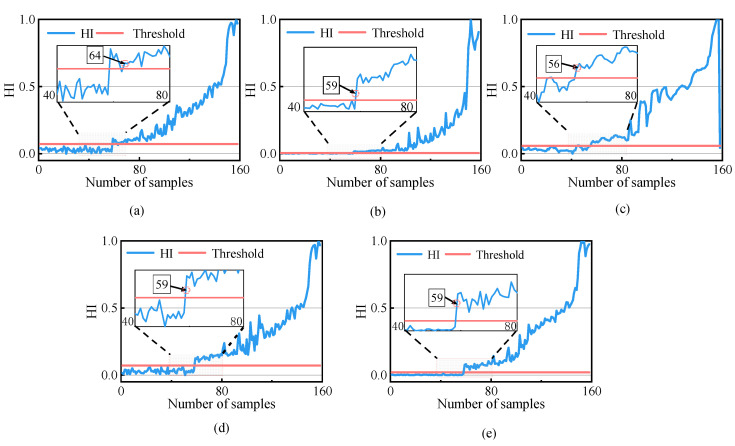
Comparison of the health indicators of different methods. (**a**) PDA results of PCA; (**b**) PDA results of RMS; (**c**) PDA results of CHMM; (**d**) PDA results of SVDD; (**e**) PDA results of the proposed methodology.

**Figure 9:**
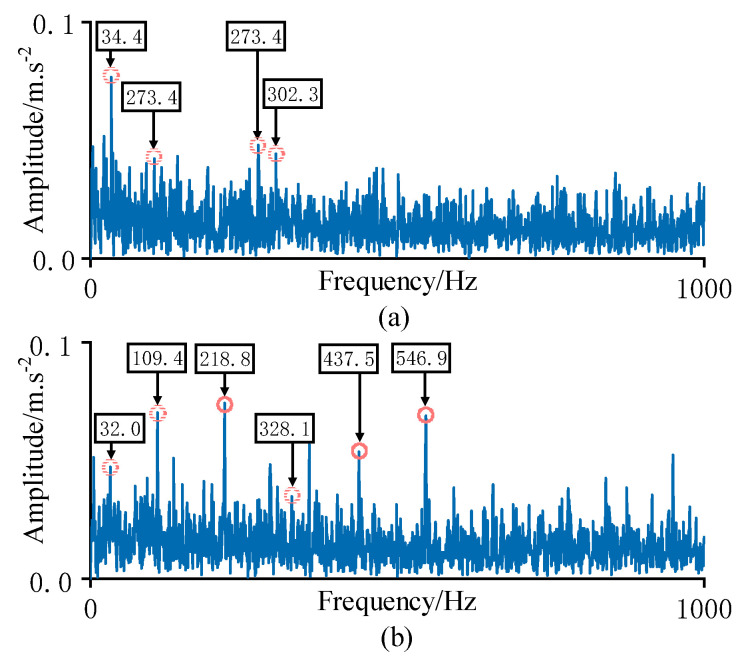
Results of envelope spectrum analysis. (**a**) Envelope spectrum of sample No. 58; (**b**) Envelope spectrum of sample No. 59.

Figure 8a–e shows the health indicators of each method. In Figure 8e, in the first 58 samples, the HI values were lower than the warning threshold, indicating that the bearing was in a healthy state. From the 59th sample onwards, the HI values exceeded the threshold and gradually increased, indicating that the rolling bearing’s performance had started to deteriorate. Envelope spectrum analysis was carried out on Samples 58 and 59, and the analytical results are shown in Figure 9. Compared to Figure 9a, the envelope spectrum in Figure 9b shows the rotation frequency of 32.0 Hz and the outer ring fault has the characteristic frequency of 109.4 Hz and its multiplier, indicating that the rolling bearing was in the healthy state before Sample 58. This shows that the proposed method accurately determined the FPT, while Method (c) determined a wrong FPT, and Method (a) determined the FPT obviously later. 

In order to quantitatively evaluate the pros and cons of HIs constructed using different methods, multiple evaluation indicators are used to evaluate the results in this study. In addition to the monotonicity index (Mon), the robustness index (Rob), and the correlation index (Cor) constructed according to Equations (1)–(3), this paper also introduces the separability index (Sep) to quantitatively evaluate the HIs. The Sep was used to measure the ability of the assessment results to discriminate the degradation stage and the ability to warn of the early failure of rolling bearings [1,30]. TOPSIS was used to combine the evaluation indicators to comprehensively measure the results of the evaluation.

The evaluation index and FPT determined using each method are displayed in Table 2. From Table 2, it can be seen that for the HI constructed using the proposed method on this dataset, only the Cor was slightly lower than the score of the CHMM method. However, the Sep, Mon, and Rob indexes had the highest score, and our proposed model had the best comprehensive score.

The local magnification of each evaluation method showed that the HI of the proposed method was the smoothest in the healthy phase, while the HI of other methods fluctuates greatly. In addition, the HI of the proposed method had the highest robustness and comprehensive score. Moreover, the envelope spectrum analysis shows that the proposed method accurately determines the FPT, which provides an adequate warning for equipment maintenance. Therefore, it can be seen that the proposed method is sensitive to the performance degradation of rolling bearings in the whole life cycle and can better tolerate outliers and false fluctuations.

### 5.2. Case 2: IEEE PHM2012 Data Challenge Dataset

#### 5.2.1. Experimental Description of Case 2

**Figure 10:**
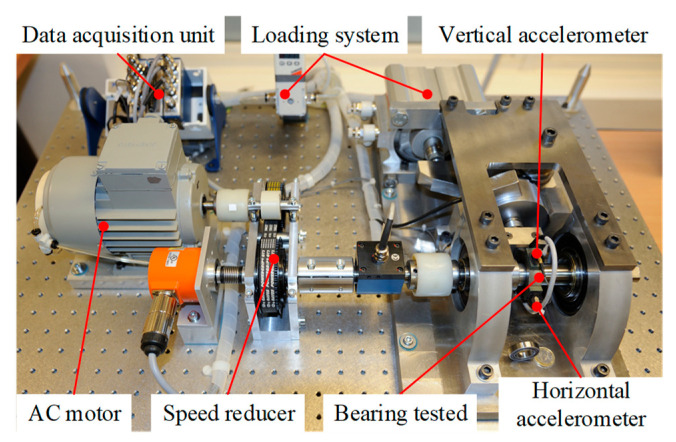
PRONOSTIA platform was used for the PHM2012 datasets.

The IEEE PHM2012 Data Challenge dataset provides the full-life vibration signals of rolling bearings in both the horizontal and vertical directions [32]. Figure 10 shows the experimental system. The sampling frequency of the vibration signal was 25.6 kHz, the sampling interval was 10 s, and the sampling time was 0.1 s.

**Figure 11:**
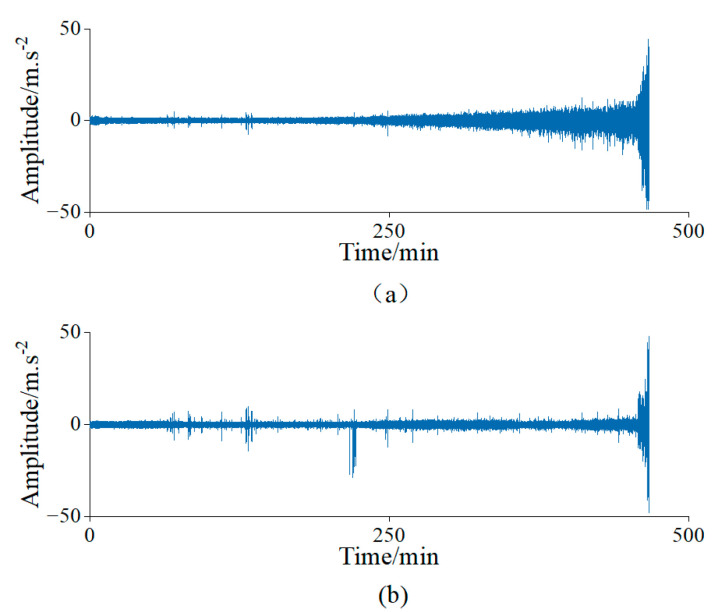
Bidirectional acceleration waveforms of rolling bearings in the time domainof Case 2. (**a**) Horizontal vibration signals; (**b**) Vertical vibration signals.

Verification was carried out using the “Bearing 1.1” subset of the data, which contained 2803 samples. Figure 11 shows the waveform diagram across the time domain for the life cycle of the bearing’s data in this test. Then, feature selection and the degradation assessment were carried out using the proposed methods.

#### 5.2.2. Experimental Result of Case 2

**Figure 12:**
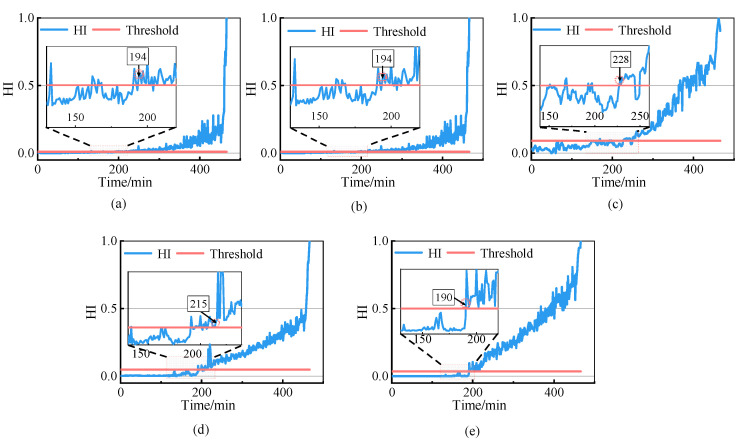
Health indicators of different methods. (**a**) PDA results of PCA; (**b**) PDA results of RMS; (**c**) PDA results of CHMM; (**d**) PDA results of SVDD; (**e**) PDA results of the proposed methodology.

The results obtained with the methods used for comparison and the proposed methods are shown in Figure 12. In Figure 12a–e, the FPT determined using the proposed method is 190 min, which is earlier than that of the other methods. In Figure 12e, in the first 190 min of operation, the HI value of most samples was below the warning threshold, indicating that the bearing was in a healthy state. After 190 min, the HI value exceeded the threshold value and increased steadily, indicating that the performance of the rolling bearings had begun to deteriorate. Since this dataset does not provide a description of the form of failure, envelope spectrum analysis was not conducted on the samples at the FPT nodes. Local magnification of each evaluation method showed that the HI of the proposed method was the most stable in the healthy phase, and the overall degradation trend was more obvious in the unhealthy state.

The indexes used for evaluating the health indicators of the previous methods for this subset of data are shown in Table 3. The comparison shows that the health indicators constructed using the proposed method in this study were optimal for all indicators and had the best overall score. 

Furthermore, the local magnification of each evaluation method shows that the HIs of this study’s method were the smoothest in the healthy phase, and the HIs of this study’s method had the highest robustness score, which shows that this method could better overcome the outliers. In addition, the HIs constructed using the proposed method had the highest comprehensive score, and it determines the FPT earlier, which shows that the proposed method could appropriately reflect the degradation of rolling bearings across their entire life cycle.

### 5.3. Case 3: Bearing Data from a Home-Made Test Bench

#### 5.3.1. Experimental Description of Case 3

**Figure 13:**
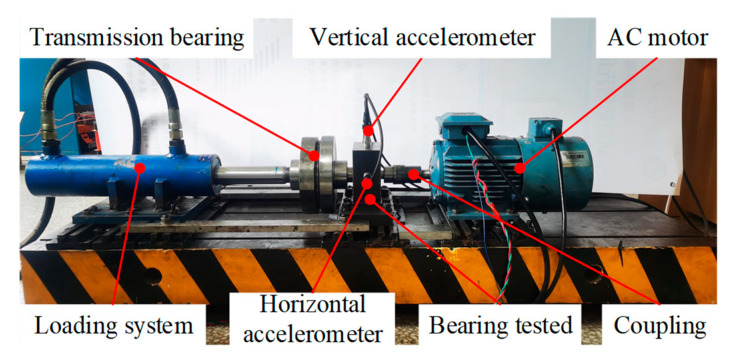
The home-made setup used for the accelerated degradation test.

In order to further verify the effectiveness of the method, experimental verification was carried out with a home-made experimental rig for testing accelerated fatigue in rolling bearings. The test bench is displayed in Figure 13. It consisted of an AC motor, a frequency converter, the coupling, the test bearing, the support bearing, a hydraulic loading system, and other components [33]. An SKF-7406 angular contact bearing was used in the experiment. During the experiment, two IMI 603C01 accelerometers were utilized to collect the vertical and horizontal vibration signals of the bearing throughout its life cycle. The sampling frequency of the vibration signal was 25.6 kHz, and the vibration signal was recorded for 1 s every 10 min. The collection was stopped when the maximum amplitude of the collected vibration signal samples exceeded 10 times the maximum amplitude of the initial sample [29].

**Figure 14:**
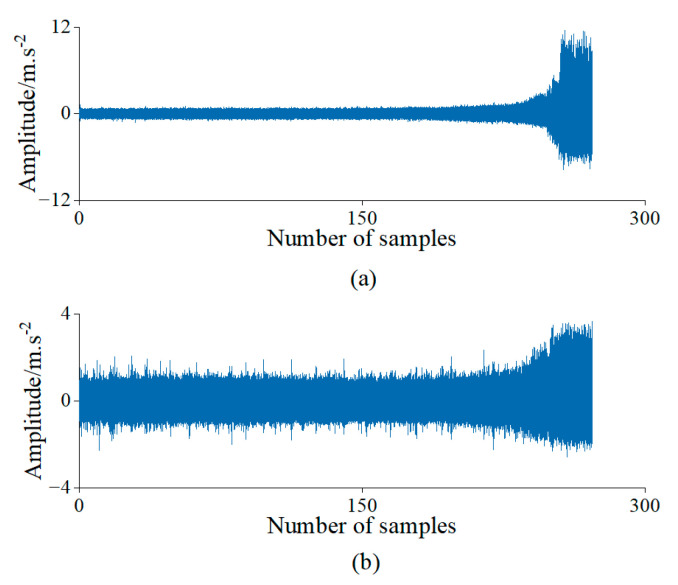
Bidirectional acceleration waveforms of rolling bearings in the time domainof Case 3. (**a**) Horizontal vibration signals; (**b**) Vertical vibration signals.

To maintain the consistency of the experimental conditions, the test bearings were run to failure under a constant load and constant speed. The vibration acceleration data collected for the bearings’ entire life cycle from the healthy state to severe degradation are shown in Figure 14. In addition, the proposed method was also used for feature selection and assessing the degradation with the data obtained from the experiments.

#### 5.3.2. Experimental Result of Case 3

**Figure 15:**
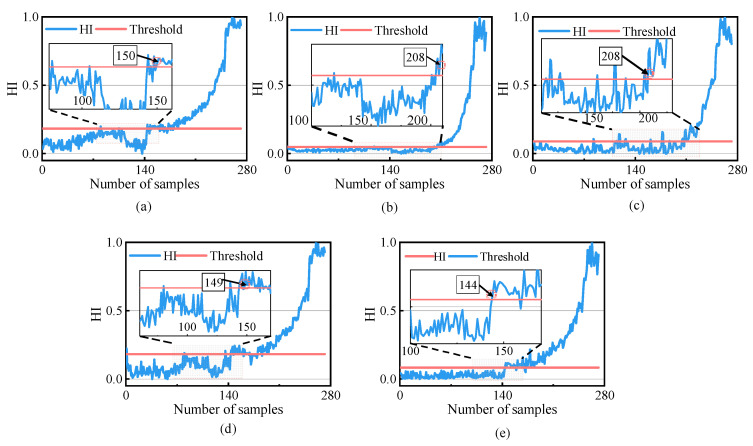
Comparison of HIs of different methods. (**a**) PDA results of PCA; (**b**) PDA results of RMS; (**c**) PDA results of CHMM; (**d**) PDA results of SVDD; (**e**) PDA results of the proposed methodology.

The results obtained using the proposed method and the methods used for comparison are presented in Figure 15, and the index values and FPT determined using each method are shown in Table 4. As can be observed in Figure 15e, during the first 145 sample periods of operation, the HI values were all below the warning threshold, indicating that the bearing was in a healthy state. After the 145th sample, the HI value continuously exceeded the threshold and gradually increased, indicating that the performance of rolling bearings had begun to deteriorate. Local magnification of each evaluation method showed that the HI of the other methods had false alarm values in the healthy phase. In contrast, the HI of this method had no false alarm values and had minimal fluctuations.

For these data, the evaluation indicators of the results of the methods mentioned above are listed in Table 4. Table 4 shows that the Cor index of the HI constructed using the proposed method was slightly lower than that of the CHMM method, but the Sep, Mon, and Rob index scores were the best, and our method had the best comprehensive score.

The proposed method had the best comprehensive score, and the FPT was determined earlier than other methods. Meanwhile, local magnification showed that the HI of this method had no false alarm values and had minimal fluctuations, and the HI had the highest robustness score, indicating that the model could better overcome the influence of outliers. Therefore, this showed a good agreement between the results of the degradation assessment and the degree of failure, accurately reflecting the health status of the bearing.

## 6. Conclusions

Determining the sensitive features set relies heavily on the prior knowledge of experts and degradation models having low-tolerance outliers and false fluctuations, and a method for evaluating the degradation of rolling bearings using adaptive sensitive feature selection and multi-strategy optimized SVDD was proposed. The effectiveness of the method was proved using experiments, leading to the following conclusions:

(1) The TOPSIS–K-medoids method was proposed for adaptive determination of the sensitive feature set. This method determines the adaptive sensitive feature set by using the monotonicity, correlation, and robustness indexes for evaluation, and the process does not need to rely on a priori knowledge to subjectively determine parameters such as the weights and thresholds, which improves the quality of the input data used for the PDA model.
(2) The multi-strategy optimized SVDD strategy trained the model using only the early samples of the healthy phase, adaptively determined the FPT, overcame the interference of outliers and false fluctuations, and better characterized the bearings’ degree of failure. The HI showed better consistency with the development trend of faults.
(3) After verification with the XJTU-SY bearing data, the IEEE PHM2012 Data Challenge dataset for bearings, and data obtained with a self-made test bench of accelerated fatigue in rolling bearings, the multi-strategy optimized SVDD model proposed in this paper demonstrated better performance compared to multiple mainstream methods according to a comparison of multiple evaluation indexes, such as monotonicity, correlation, robustness, and separability.

In summary, a rolling bearing performance degradation assessment method with the combination of adaptive sensitive feature selection and multi-strategy optimized SVDD was proposed in this paper. The proposed feature selection method determines the adaptive sensitive feature set with multiple feature evaluation indexes instead of prior knowledge; the multi-strategy optimized SVDD only uses the early samples in the healthy stage to train the model and adaptively determines the FPT while better overcoming the interference of outliers and false fluctuations. The proposed model could accurately reflect the degradation status of rolling bearings verified using experiments, which has a positive effect on the early detection of potential failure of rolling bearings and their maintenance.

## Figures and Tables

**Table 1:** Time domain and frequency domain features.

Name	Equation	Name	Equation
Mean value	$\mu_{s}=\frac{1}{n}(\sum_{i=1}^{n}s_{i})$	Standard deviation	$\sigma_{s}=\sqrt{\frac{1}{n-1}\sum_{i=1}^{n}{\left(s_{i}-\mu_{S}\right)}^{2}}$
Average amplitude	$\mu_{a}=\frac{1}{n}\sum_{i=1}^{n}|s_{i}|$	Variance	$\sigma_{s}^{2}=\frac{1}{n-1}\sum_{i=1}^{n}{\left(s_{i}-\mu_{S}\right)}^{2}$
Maximum value	$F_{\max}=\max(s_{i})$	Kurtosis	$F_{kurt}=\frac{1}{n}\sum_{i=1}^{n}{\left(s_{i}-\mu_{S}\right)}^{4}$
Minimum value	$F_{\min}=\min(s_{i})$	Skewness	$F_{SK}=\frac{1}{n}\sum_{i=1}^{n}{\left(s_{i}-\mu_{S}\right)}^{3}$
Peak value	$F_{\left|\max\right|}=\max(|s_{i}|)$	Waveform index	$F_{WI}=F_{RMS}/\mu_{a}$
Peak to peak value	$F_{P2P}=F_{\max}-F_{\min}$	Peak index	$F_{PI}=F_{\left|\max\right|}/F_{RMS}$
Root mean square	$F_{RMS}=\sqrt{\frac{1}{n}\sum_{i=1}^{n}s_{i}{}^{2}}$	Impulse index	$F_{IF}=F_{\left|\max\right|}/\mu_{a}$
Root amplitude	$F_{RA}={\left(\frac{1}{n}\sum_{i=1}^{n}\sqrt{\left|s_{i}\right|}\right)}^{2}$	Tolerance index	$F_{MF}=F_{\left|\max\right|}/F_{RA}$
Kurtosis index	$F_{KI}=\frac{\sum_{i=1}^{n}{\left(s_{i}-\mu_{S}\right)}^{4}}{(n-1)\sigma_{s}{}^{4}}$	$F_{1}$	$\mu_{sk}=\frac{1}{n}\sum_{i=1}^{n}sk_{i}$
$F_{2}$	$\sigma_{sk}=\sqrt{\frac{1}{n-1}\sum_{i=1}^{n}{\left(sk_{i}-\mu_{Sk}\right)}^{2}}$	$F_{3}$	$F_{3}=\sqrt{\frac{\sum_{i=1}^{n}{\left(sk_{i}-\mu_{Sk}\right)}^{3}}{n\sqrt{\sigma_{sk}{}^{3}}}}$
$F_{4}$	$F_{4}=\sqrt{\frac{\sum_{i=1}^{n}{\left(sk_{i}-\mu_{Sk}\right)}^{4}}{n\sigma_{sk}{}^{2}}}$	$F_{5}$	$F_{5}=\frac{1}{n}\sum_{i=1}^{n}f_{i}$
$F_{6}$	$F_{6}=\sqrt{\frac{1}{n}\sum_{i=1}^{n}{\left(f_{i}-F_{22}\right)}^{2}sk_{i}}$	$F_{7}$	$F_{7}=\sqrt{\frac{\sum_{i=1}^{n}f_{i}{}^{2}sk_{i}}{\sum_{i=1}^{n}sk_{i}{}_{i}}}$

**Table 2:** Evaluation results of the different health indicators.

Index Method	FPT	Sep	Mon	Cor	Rob	TOPSIS Score
PCA	64	0.703	0.981	0.828	0.989	0.621
RMS	59	0.370	0.974	0.801	0.773	0.325
CHMM	56	0.563	0.912	0.953	0.898	0.532
SVDD	59	0.523	0.967	0.824	0.881	0.477
Proposed method	59	0.900	0.986	0.927	0.922	0.914

**Table 3:** Evaluation of the different health indicators.

Index Method	FPT	Sep	Mon	Cor	Rob	TOPSIS Score
PCA	194	0.406	0.965	0.608	0.830	0.097
RMS	194	0.318	0.962	0.608	0.890	0.201
CHMM	228	0.671	0.981	0.955	0.951	0.821
SVDD	215	0.518	0.969	0.866	0.958	0.567
Proposed method	190	0.812	0.987	0.959	0.962	0.998

**Table 4:** Evaluation of the different health indicators.

Index Method	FPT	Sep	Mon	Cor	Rob	TOPSIS Score
PCA	150	0.660	0.964	0.898	0.892	0.626
RMS	208	0.535	0.962	0.859	0.812	0.407
CHMM	208	0.582	0.917	0.933	0.864	0.500
SVDD	149	0.602	0.927	0.863	0.884	0.519
Proposed method	144	0.671	0.965	0.884	0.892	0.835

## Data Availability

The data presented in this study are available on request from the corresponding author.
